# Photoaffinity Labeling of Plasma Proteins

**DOI:** 10.3390/molecules181113831

**Published:** 2013-11-08

**Authors:** Victor Tuan Giam Chuang, Masaki Otagiri

**Affiliations:** 1 School of Pharmacy, Faculty of Health Sciences, Curtin Health Innovation Research Institute, Curtin University, GPO Box U1987, Perth 6845, WA, Australia; 2 Graduate School of Pharmaceutical Sciences, DDS Research Institute, Sojo University, Kumamoto 860-0082, Japan

**Keywords:** photoaffinity labeling, plasma proteins, human serum albumin, α_1_-acid glycoprotein, photoreactivity, protein binding, labeling specificity

## Abstract

Photoaffinity labeling is a powerful technique for identifying a target protein. A high degree of labeling specificity can be achieved with this method in comparison to chemical labeling. Human serum albumin (HSA) and α_1_-acid glycoprotein (AGP) are two plasma proteins that bind a variety of endogenous and exogenous substances. The ligand binding mechanism of these two proteins is complex. Fatty acids, which are known to be transported in plasma by HSA, cause conformational changes and participate in allosteric ligand binding to HSA. HSA undergoes an N-B transition, a conformational change at alkaline pH, that has been reported to result in increased ligand binding. Attempts have been made to investigate the impact of fatty acids and the N-B transition on ligand binding in HSA using ketoprofen and flunitrazepam as photolabeling agents. Meanwhile, plasma AGP is a mixture of genetic variants of the protein. The photolabeling of AGP with flunitrazepam has been utilized to shed light on the topology of the protein ligand binding site. Furthermore, a review of photoaffinity labeling performed on other major plasma proteins will also be discussed. Using a photoreactive natural ligand as a photolabeling agent to identify target protein in the plasma would reduce non-specific labeling.

## 1. Introduction

Identification of target proteins is pivotal in drug discovery, and elucidation of the ligand binding site topology on the target protein is critical for drug development. Photoaffinity labeling is a useful and reliable method to identify the binding protein in complex mixtures of biological molecules such as those found in the cytosol or the plasma. Through this method, structural information on the ligand binding site can be obtained by identifying the amino acid residues that are located at these sites. Thus, photoaffinity labeling is a powerful tool that is applicable in both drug discovery and development processes [1].

**Figure 1:**
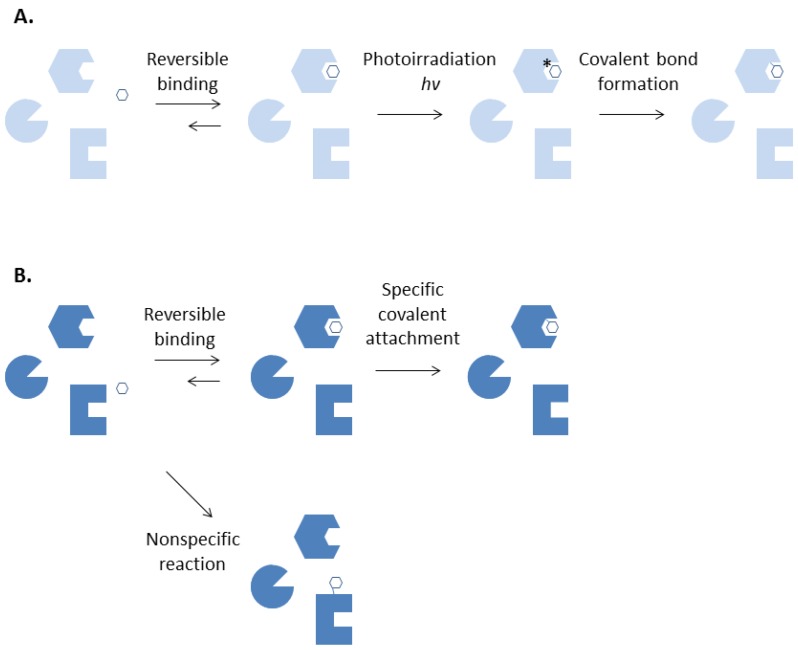
Photoaffinity labeling (**A**) and chemical affinity labeling (**B**).

Different from chemical labelling, photoaffinity labeling enables formation of a covalent bond between a ligand and its specific binding protein at will. (Figure 1) The technique requires a chemically inert ligand to be converted into a highly reactive intermediate by irradiation with near ultraviolet or visible lights after its binding to a protein is complete. This will allow important preliminary experiments of a photoaffinity labeling agent such as binding affinity measurements and assays of biological activities to be performed with ease [2]. Covalent adduct formation can be confined to the desired site, thereby further reduce non-specific labeling, by removing the unbound photolabeling reagents after incubation and rapidly irradiate the ligand-protein complex before dissociation takes place.

The photogenerated species are usually so reactive that they can react with carbon-hydrogen bonds. This precludes the need for the presence of a particular functional group at the binding site of the protein for the photogenerated intermediates to react and form a covalent bond with. In contrast, functional group-specific reagents may be essential in a chemical affinity labeling experiment in order to form a covalent bond with a specific functional group that is present at the protein binding site. Extreme conditions that are detrimental to the proteins such as extreme pH values, high temperatures or pre-reduction of disulfide bonds may be required for efficient reaction of a chemical labeling reagent. Misleading results can be obtained when attempts are made to chemically label an inert binding site lacking of the required functional group. Furthermore, keeping the non-specific labeling at an acceptable low level is a constant challenge in chemical labeling experiments [3].

The major drawbacks of photoaffinity labeling experiments is the photooxidative damage to proteins at short wavelengths due to the direct reaction of excited chromophores such as tryptophan in the protein with oxygen, or because of the generation of oxygen radicals by the photolabeling agent in the sample. Some photolabeling agents may cause problems at wavelengths in the visible region. Photooxidative damages include crosslinking of proteins and the loss of binding capacity of proteins for ligands. One way of overcoming this problem is to pass a stream of water-saturated nitrogen or argon to remove dissolved oxygen in order to reduce the level of such photooxidative damage [3].

**Figure 2:**
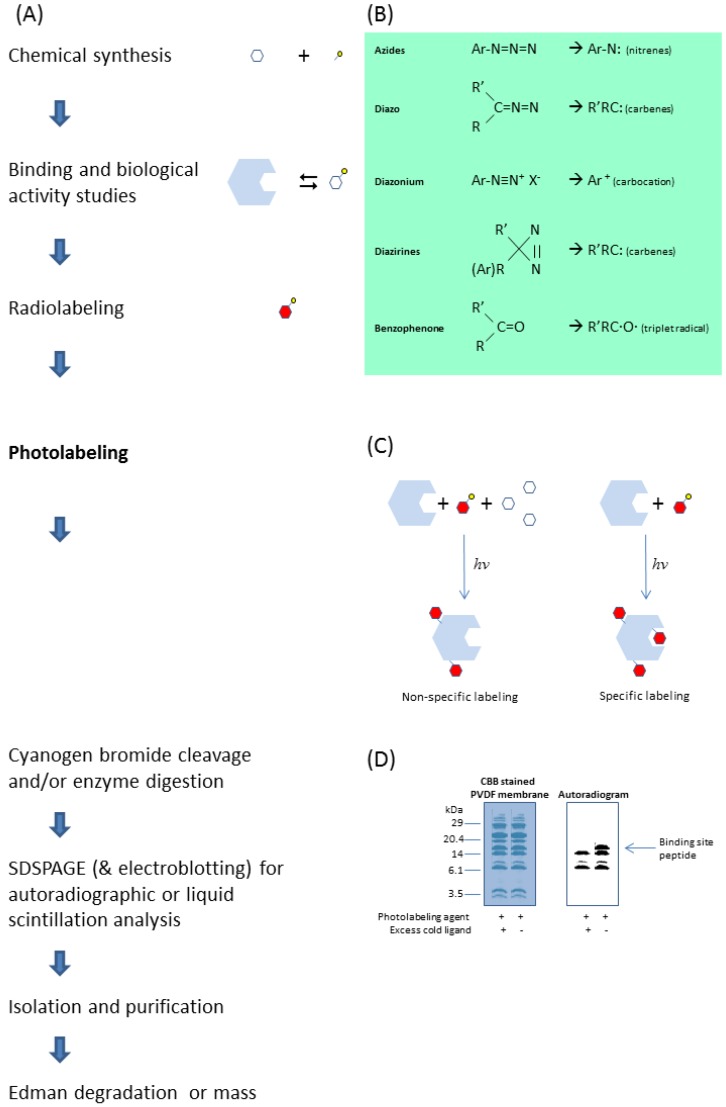
A typical photoaffinity labeling experimental protocol to identify the ligand binding site of a target protein (**A**), starting from synthesizing the photolabeling agent by linking the ligand with a suitable photoreactive moiety (**B**). Photolabeling the protein in the presence of excess parent ligand (**C**) would allow identification of the binding site peptide from the non-specifically labeled peptides of the protein after chemical cleavage or enzymatic digestion (**D**) [2,3,6].

Photoaffinity labeling can be a powerful technique for studying the properties and functions of a protein. Figure 2 summarizes a scheme of a typical photoaffinity labeling experimental protocol for identification of the ligand binding site amino acid sequence. A direct link can then be made by site-directed mutagenesis of the amino acid residues that were previously identified by photoaffinity labeling. Depending on the nature of the mutation, various functional changes including ligand binding properties may take place, which can be reconfirmed by photoaffinity labeling of the mutants. Identifying amino acid residues for which mutations alter the functional properties of the biological protein allow structure function relationships to be evaluated and interpreted.

## 2. Plasma Proteins

Plasma proteins comprise a variety of proteins that can be classified according to their known roles and functions. They include immunoglobulins, hormones, cytokines, blood coagulation and fibrinolysis, complements, enzymes, lipoproteins, transport and storage proteins, and other proteins [4]. Human serum albumin (HSA) belongs to the class of transport and storage proteins whereas α_1_-acid glycoprotein (AGP) is classified under immune systems, since it is an acute phase protein. They are two major plasma proteins that are known to bind a broad spectrum of drugs, in addition to their extraordinary binding capacity for endogenous substances [5]. There are proteins of other classes that also interact with a great variety of yet to be identified endogenous as well as exogenous substances. In addition to understanding the physiological role and function of each of these plasma proteins, knowledge of the range of their interacting ligands will certainly foster and enhance drug discovery and development processes. 

### 2.1. Human Serum Albumin

**Figure 3:**
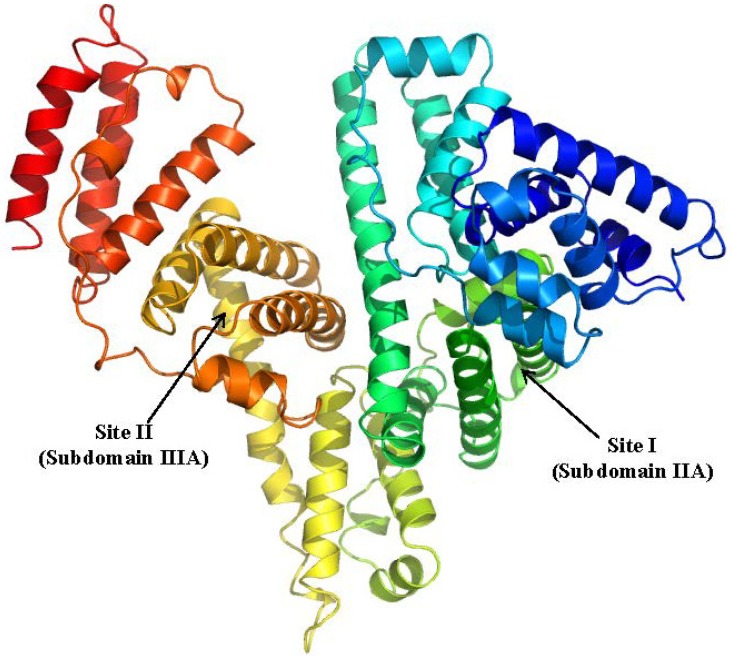
Crystal structures of the human serum albumin. The illustration was produced by PyMol using the atomic coordinates from Protein Data Bank, PDB2BXA [9].

HSA is the most abundant protein in plasma that is responsible for maintaining the oncotic pressure of blood vessels. (Figure 3) It is also a major carrier of a broad spectrum of endogenous substances such as long chain fatty acids, bilirubin and thyroid hormones. HSA has two major and distinct sites for the high-affinity binding of several physiologically important compounds and a large number of drugs, namely site I and site II [7,8]. Crystallographic investigations found that drug binding sites I and II are located in subdomains IIA and IIIA respectively [9]. Ligands that interact with site I include thyroxine, bilirubin, warfarin whereas diazepam, octanoic acid, arylpropionic acids such as ketoprofen (KP) and ibuprofen primarily bind to site II.

#### 2.1.1. Ketoprofen

**Figure 4:**
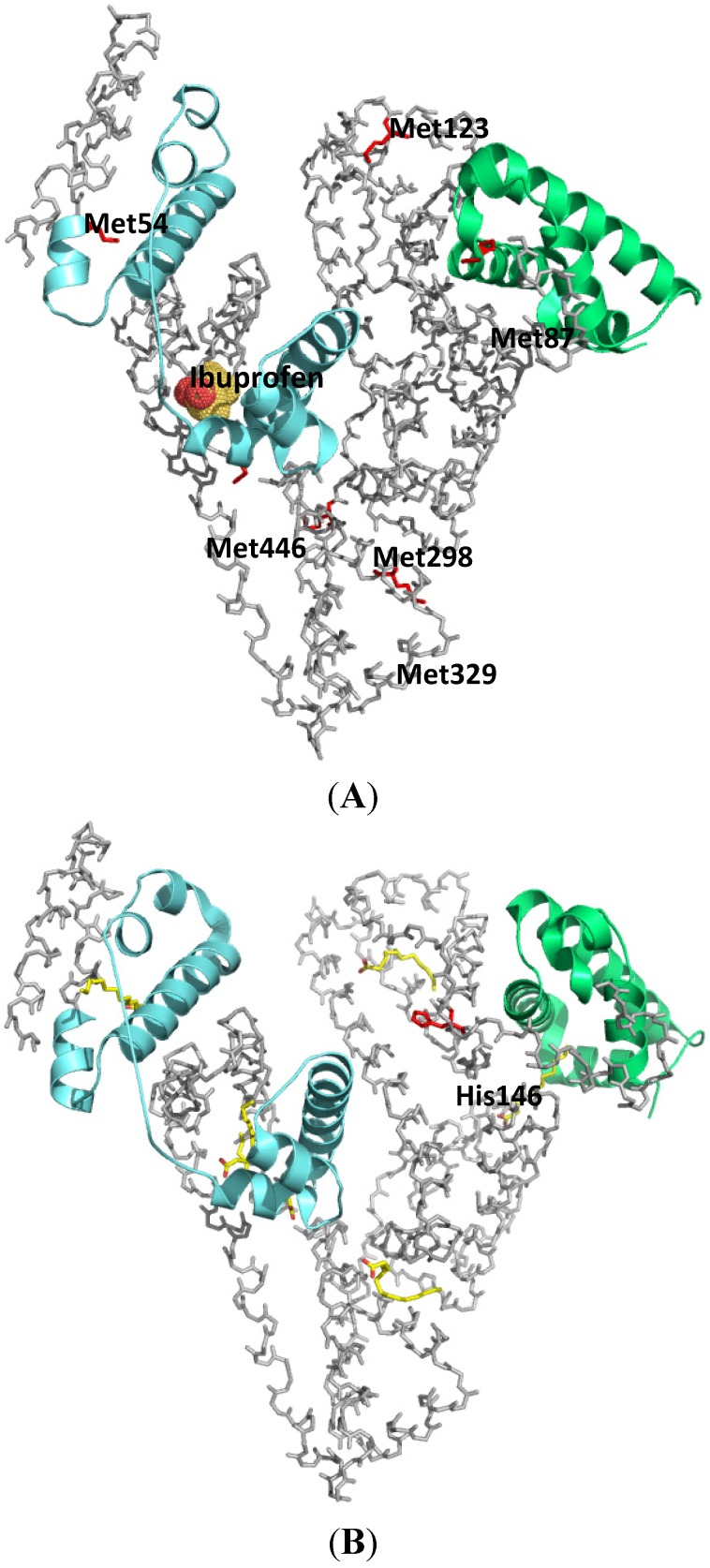
Crystal structures of HSA showing the locations of methionine residues (**A**), histidine146 and the binding sites of myristates (**B**). The 9.4 kDa (subdomain IA) and 11.6 kDa (subdomain IIIA) CNBr fragments photolabeled by [^14^C]KP were shown in green and cyan respectively. Both illustrations were produced by PyMol using the atomic coordinates from the Protein Data Bank, 2BXG for (A), 2BXL for (B).

KP has a photoreactive benzophenone moiety in its chemical structure [10], hence it can be used as a photoaffinity labeling reagent without further structural modification. Cyanogen bromide cleavage of the HSA photolabeled with [^14^C]KP revealed that 11.6 kDa and 9.4 kDa fragments, corresponding to domain III and subdomain IA respectively, contain most of the photoincorporated radioactivity. (Figure 4A) Competition experiments using other site II ligands, diazepam (DZP) and octanoic acid, inhibited the photobinding of [^14^C]KP to the 11.6 kDa fragment, suggesting that this fragment contains the common binding region for site II ligands [11]. Further photolabeling studies with [^14^C]KP on native and recombinant mutants albumins, R410C and R485A confirmed the importance of R410 in the high affinity binding of arylpropionic acid derivatives possibly through electrostatic interaction, whilst R485 contributed to the binding in the form of non-electrostatic interaction with the benzophenone moiety of KP since R485 was found to form a covalent adduct with KP [12].

Long chain fatty acids are insoluble in water and are carried in the plasma either in the form of esterified triacylglycerols carried by lipoproteins, or in a non-esterified form bound to albumin. The plasma concentration of HSA is around 0.6 mM and the molecule can carry at least six molecules of fatty acids. Under normal physiological conditions, HSA carries approximately 0.1–2 moles of fatty acids per mole of protein. No significant increase in the free fraction of KP was observed when an ultrafiltration binding experiment was performed in the presence of up to a myristate (MYR):HSA ratio of 3. Based on the results of ultrafiltration experiments, it is reasonable to conclude that the first three molecules of MYR bind to a different region than subdomain III. Photolabeling of HSA with [^14^C]KP in the presence of MYR at increasing MYR:HSA ratios of 1 to 3 showed a decrease in [^14^C]KP covalent binding to subdomain III but an increase in the binding to subdomain IA. The results of photoaffinity labeling revealed that a concurrent competitive and allosteric binding mechanism by MYR up until a MYR:HSA ratio of 3 is operative, which mainly involves domain I as the site for allosteric binding of the displaced [^14^C]KP. This illustrates the usefulness of the photoaffinity labeling technique in examining the complex binding behavior of HSA especially when long-chain fatty acids are involved (Figure 4B) [13].

HSA undergoes a conformational change at different pH values, which appears to have an impact on its ligand binding properties [5,14]. Yamasaki *et al*. reported that when HSA undergoes a conformational change from the neutral (N) to the base (B) conformation (the N-B transition) in the physiological pH region, a “competitive”-like strong allosteric regulation exists that affects both of the major binding, sites I and II, of HSA [15]. The results of ultrafiltration binding experiments reported by Kaneko *et al*. performed at increasing pH of 6.5, 7.4 and 8.2 indicated an increase in the percentage binding of KP to HSA with increasing pH. Interestingly, the results of photoaffinity labeling studies performed at similar pH conditions showed a comparable extent of covalent binding of [^14^C]KP to domain III but there was an increase in the covalent binding of [^14^C]KP to subdomain IA was observed with increasing pH [16]. In a previous study, Kosa *et al*. proposed that histidine residues located in domain I were responsible for the N-B transition [17]. In a point mutation study, Yang *et al*. confirmed that H9, H67, H105, H128 and H146 are significant contributors to the N-B transition via electrostatic interactions [18]. In an attempt to examine the role of histidines in N-B transition induced ligand binding change, Kaneko *et al*. photolabeled six histidine single mutants of HSA, namely, H9A, H39A, H67A, H105A, H128A and H146A, with [^14^C]KP at pH 6.5, 7.4 and 8.2 and concluded that H146 plays a prominent role in the allosteric binding induced by N-B transition [16].

#### 2.1.2. Flunitrazepam

Site II of HSA, also known as the indole-benzodiazepine (BDZ) site, is a primary binding site for DZP and other BDZ drugs. However, differences in the binding characteristics of BDZ drugs that contain an asymmetric center as well as the type of moiety at position 7 have been reported. Noctor *et al*. reported the existence of a very low affinity nitro-BDZ site, or sites that may be different from site II [19,20]. Flunitrazepam (FNZP) is a BDZ with a nitro group at position 7 that did not displace the site II fluorescent probe, dansylsarcosine, to an extent similar to that of DZP. FNZP has been used as a photolabeling agent in BDZ-receptor studies [21]. The photolabeling profiles of HSA with [^3^H]FNZP in the presence of site I and site II ligands, including DZP, indicated that these ligands did not inhibit the covalent binding of [^3^H]FNZP to HSA. In contrast, DZP inhibited the covalent binding of [^3^H]FNZP to AGP. These results suggest that subdomain III A of HSA does not function as a binding site for FNZP [22].

#### 2.1.3. Halothane

In an attempt to investigate the interaction of volatile anesthetics and proteins, Eckenhoff *et al*. photolabeled bovine serum albumin (BSA) with [^14^C]halothane in the presence and absence of other volatile anaesthetics by exposing the mixture to 254 nm UV light for 10 s. Chloroform, methoxyflurane, isoflurane, diethyl ether, ethanol, oleate and low pH inhibited photoincorporation of [^14^C]halothane to BSA [23]. In a subsequent photolabeling experiment to determine the binding site amino acid sequence, BSA was found to have a higher affinity for [^14^C]halothane than HSA. The major photolabeled site of [^14^C]halothane was in subdomain IIA involving tryptophan residue for both BSA and HSA [24]. Two molecules of halothane were found to bind at the interface between subdomains IIA and IIB, in close proximity with tryptophan 214, which can also bind a fatty acid molecule in an X-ray crystallographic study conducted by Battacharya *et al*. [25].

#### 2.1.4. Leukotriene

Albumin has been reported having a stabilising effect on leukotriene A4 that is reversed by warfarin which binds to subdomain IIA (site I) [26]. On the other hand, leukotriene B4 has been reported to bind to subdomain IIIA (site II) [27]. Falk *et* *al*. showed that albumin was labelled when rat as well as human serum was subjected to direct photoaffinity labeling in the frozen state after cryofixation using [^3^H_8_]leukotriene E4, followed by irradiation at 300 nm. Cryofixation eliminates the unspecific labeling by photoisomers and photodegradation products of leukotrienes [28]. However, no attempt has been made to characterise the binding site of leukotriene E4 on the albumins.

### 2.2. α_1_-Acid Glycoprotein

**Figure 5:**
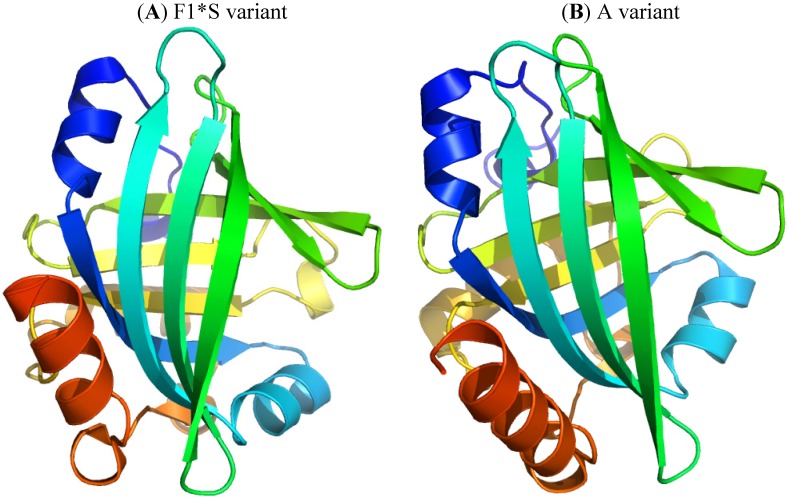
Crystal structures of the human AGP F1*S (**A**) and A variants (**B**). Both illustrations were produced by PyMol using the atomic coordinates from the Protein Data Bank, 3KQ0 for (A) [37] and 3APX for (B).

α_1_-Acid glycoprotein (AGP), or orosomucoid, is a member of the lipocalin family. It is classified as an acute phase protein, since its serum concentration increases from 2- to a 10-fold in response to various pathophysiological conditions such as a systemic tissue injury, an inflammation or an infection. AGP is a single chain glycoprotein with a molecular weight of 41–43 kDa and contains 183 amino acid residues with two disulfide bridges. It is heavily glycosylated, with a carbohydrate content that accounts for 45% of the molecular weight, and it is negatively charged, with a pI of 2.8–3.8 due to the presence of sialic acid residues. Five highly sialylated complex-type-N-linked glycans are attached to asparagine residues 15, 38, 54, 75 and 85. The microheterogeneity of AGP’s glycosylation can be modified and change depending on the type of systemic inflammation or disease. In addition, plasma AGP is a mixture of genetic variants, consisting of at least two genetic variants, namely, F1*S (Figure 5A) and A variants (Figure 5B), the relative proportions of which change during an acute phase reaction such as inflammation. The appearance of different glycosylation patterns of AGP can influence its immunomodulatory and ligand binding properties. AGP binds numerous basic and neutral lipophilic drugs as well as endogenous steroid hormones such as progesterone. AGP can also bind acidic drugs such as phenobarbital [29,30,31,32,33,34,35,36]. Each genetic variant of AGP has a different ligand binding specificity that contributes to the complexity of ligand binding to AGP [37].

#### 2.2.1. Flunitrazepam

Further investigations of the binding site of FNZP in AGP through photoaffinity labeling revealed that sialic acid is not involved in the binding and that FNZP is a common ligand between the F1*S variants and A variant. The photolabeled peptide corresponded to Tyr91-Arg105 of AGP [38]. In addition to FNZP, clonazepam has been reported to be a photoaffinity labeling agent by Sieghart *et al*. [39].

#### 2.2.2. 7-Hydroxystaurosporine (UCN-01)

7-Hydroxystaurosporine (UCN-01) is a protein kinase inhibitor anticancer drug that exhibits an extraordinary high binding affinity for AGP involving tryptophan residues of AGP. Like FNZP, no significant difference was found for the binding of UCN-01 to F1*S and A variants of AGP [40]. The photolabeling of AGP with UCN-01 indicated that Trp-160 formed a covalent adduct with the drug. This result was confirmed by the finding that only the mutant W160A of AGP showed a marked decrease in the extent of photoincorporation in another photolabeling study on three recombinant AGP mutants (W25A, W122A, and W160A) [41].

#### 2.2.3. 3-(2-(3-Azido-4-iodophenylpropionamido)-ethoxy)thalidomide

Turk *et al*. synthesized a photoactive thalidomide derivative as a probe to elucidate the molecular mechanism for the immunomodulatory effect of thalidomide, which has been attributed to the selective inhibition of tumor necrosis factor alpha from monocytes. A pair of proteins of 43–45 kDa with a high acidity from bovine thymus extract were photolabeled with the photoactive thalidomide derivative and found to be AGP. A subsequent photolabeling study of human AGP revealed that thalidomide inhibited the covalent binding at therapeutic concentrations for inhibition of tumor necrosis factor alpha production from human monocytes, suggesting that AGP may be involved in the immunomodulatory activity of thalidomide [42].

### 2.3. Thyroxine-Binding Prealbumin (Transthyretin)

**Figure 6:**
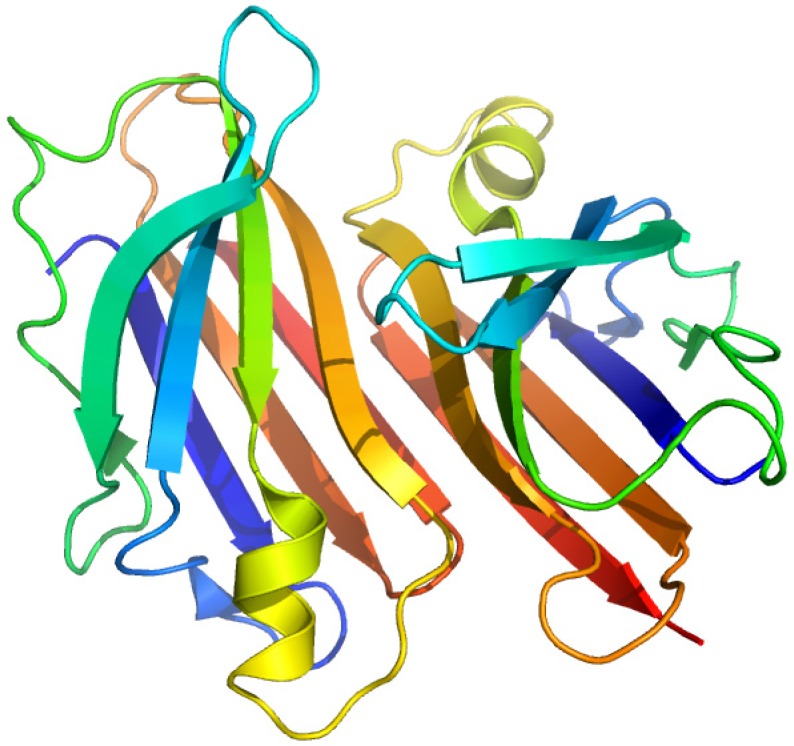
Crystal structures of the human transthyretin. The illustration was produced by PyMol using the atomic coordinates from the Protein Data Bank, PDB1DVQ [46].

Thyroxine-binding prealbumin (transthyretin, TTR) is a protein located in the “prealbumin” zone upon an electrophoretic analysis of plasma proteins. (Figure 6) It is synthesized in the liver, choroids plexus in the brain, and the eye. Its binding affinity for thyroxine is less than that of thyroxine-binding globulin but greater than that of albumin. It also transports retinol via binding to the retinol-binding protein, hence the name trans(port)-thy(roxine)-retin(ol). TTR exists in homotetramer that complexes with the retinol-binding protein in a molar ratio of 1:1 in plasma. TTR has a dimer configuration with a central channel containing two high-affinity binding sites for thyroid hormones. Anthranilic acid derivatives, such as flufenamic, meclofenamic, and mefenamic acids, have also been found to interact strongly with TTR [43]. The highly conserved structure of TTR has been shown to be associated with the pathogenesis of Alzheimer’s disease, depression, and lead toxicity. Since TTR is a highly amyloidogenic protein because it contains a β-sheet structure, it becomes a precursor protein in familial amyloidotic polyneuropathy. Moreover, TTR plays important roles in various central nervous system disorders, diabetes mellitus, and lipid metabolism [44,45].

#### 2.3.1. [3'5'-^125^I]-Thyroxine and Triiodothyronine

Dozin *et al*. reported that irradiation with light above 300 nm photolabeled [3',5'-^125^I]thyroxine (T4) or [3'-^125^I]triiodothyronine (T3) to human thyroxine binding globulin and BSA [47]. Van der Walt *et al*. reported that the irradiation of T4 and 3,5,3'-T3 with near UV (wavelengths greater than 300 nm) for 30 min and 18 h resulted in the covalent binding to albumin and TTR respectively. The mechanism of labelling was proposed to be due to homolytic fission of C-I bonds, which results in the generation of phenyl radicals, and possibly also to iodine radicals. The absence of aromatic residues at the binding site of TTR as well as the low degree of freedom of movement of T4 within the binding site contributed to the slow covalent bond formation with TTR [48]. On the other hand, Somack *et al*. demonstrated that the photoirradiation of TTR at 254 nm for a much shorter length of time, 15 s, with [^125^I]-L-T4 also led to covalent bond formation, and the same extent of photolabeling could be achieved after 25 min of irradiation with a 350 nm light source. It is noteworthy that since the T4 used in the two studies were radiolabeled with ^125^I exclusively in the outer ring 3’ and 5’ positions, if some of the covalent bond formation involved the photoinduced loss of these iodines, then the specific radioactivity of the covalently bound ligand would be reduced thus leading to an underestimation of the extent of photoattachment. This might be the reason for the increase in the amount of covalently bound radioactivity to HSA from 25% to 46% when the phenolic ring-labeled [3’,5’-^125^I]T4 was replaced with side chain-labeled [2-^14^C]T4 reported by van der Walt [48].

#### 2.3.2. Thyroxine Derivatives

^125^I-Labeled 3-[4-(4-hydroxy-3,5-diiodophenoxy)-3,5-dinitrophenyl]-propionic acid has been shown to be able to form covalent attachment to TTR upon photoirradiation at 317 nm for 1 h [49]. On the other hand, Somack *et al*. synthesized N-(ethyl-2-diazomalonyl)-3,5,3'-triiodothyronine (EDM-T3) and N-(ethyl-2-diazomalonyl)thyroxine (EDM-guT4) as thyroid hormone analogues to photolabel TTR, T4 binding globulin, and albumin in unfractionated human serum. The photolysis of [^125^I]-L-EDM-T4 and TTR mixture at 254 nm resulted in the covalent binding of [^125^I]-L-EDM-T4 to TTR. The mechanism of covalent photoattachment was proposed to occur via a carbene-dependent pathway derived from the photolysis of the diazo group, and a carbene-independent pathway that may involve attachment via radical formation following the photoinduced loss of the thyronine ring iodine. The efficiency of photoattachment of [^125^I]-L-EDM-T4 to TTR was concluded to be higher than L-T4 [50].

### 2.4. Plasma Retinol-Binding Protein

**Figure 7:**
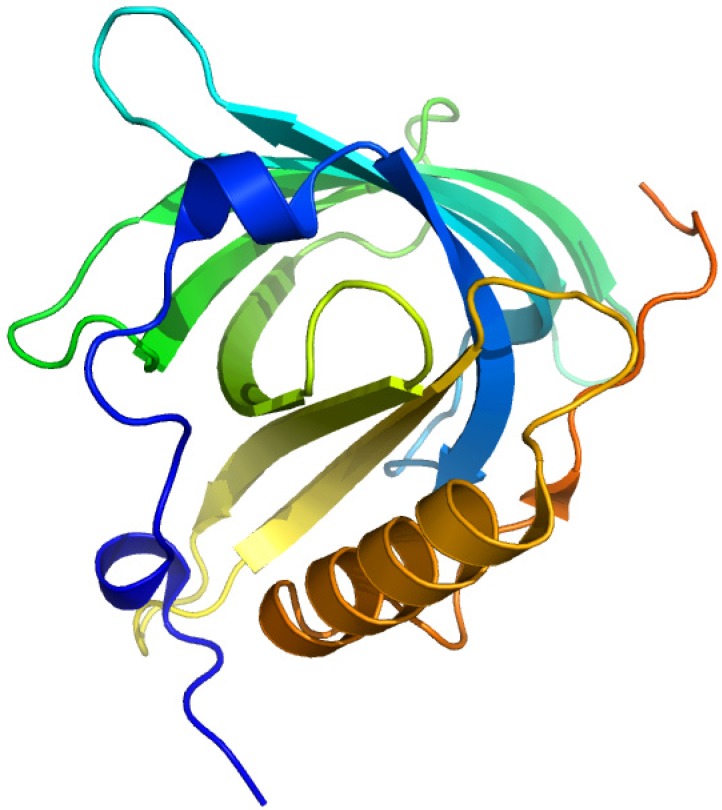
Crystal structures of the human plasma retinol binding protein. The illustration was produced by PyMol using the atomic coordinates from the Protein Data Bank, PDB1JYD [54].

**Figure 8:**
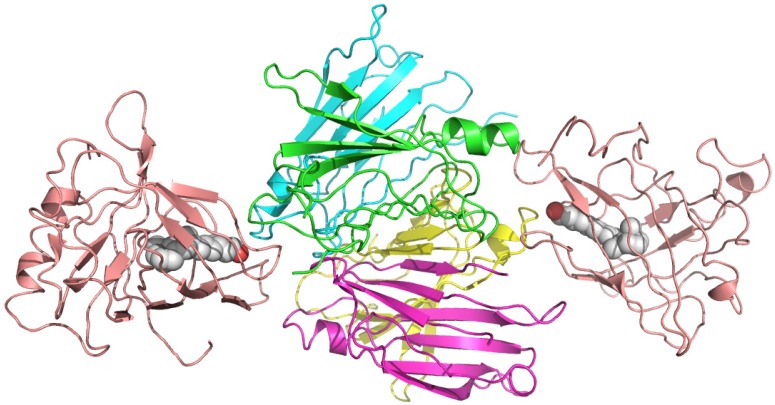
Crystal structures of the hexameric complex of human retinol-binding protein (RBP) with its carrier protein transthyretin. The RBP molecules are shown in salmon red, one of the TTR dimers is in green and yellow, and the other is in blue and magenta. The retinol molecules are represented as space-filling models with white carbon atoms and a hotpink oxygen. The illustration was produced by PyMol using the atomic coordinates from the Protein Data Bank, PDB 1QAB [55].

Plasma retinol-binding protein (RBP) is a member of the lipocalin family that specifically carries all-trans-retinol in the blood, delivering retinol to peripheral tissues from stores in the liver. (Figure 7) RBP itself is bound to TTR in a 1 to 1 stoichiometry when circulating in the plasma. *In vitro*, one tetramer of TTR can bind two molecules of retinol-binding protein. (Figure 8) [51]. The TTR and RBP complex is an absolute requirement for retinol binding, but a RBP containing retinoic acid does not form complex with TTR [52]. This protein-protein complexation between the RBP and TTR prevents RBP from being filtered and loss via kidney glomeruli. Within the cell, the all-trans-retinol molecule, as well as its oxidized form all-trans-retinal, is bound to one of the two isoforms of cellular retinol-binding proteins (CRBP I and CRBP II), while the oxidized form of all-trans-retinal, all-trans-retinoic acid binds to one of the two cellular retinoic acid binding protein isoforms (CRABP I and CRABP II) [53].

#### 2.4.1. All-*Trans*-Retinoic Acid and Azidoretinoids

Retinoic acid can be used as a photoaffinity probe without further modification due to the fact that it contains an α,β-unsaturated carbonyl group and has a highly conjugated polyene structure. After exposure of the radioactively labeled all-trans-retinoic acid in complex mixtures of cytosolic proteins to an intense 365 nm UV light for 15 min, two known retinoic acid-binding proteins, CRBP and albumin, were labelled specifically [56]. Direct photoaffinity labeling of cellular retinoic acid-binding protein I with all-*trans*-retinoic acid identified Trp7, Lys20, Arg29, Lys38, and Trp109 to be the amino acids that have been modified [57]. On the other hand, C(5)-azido-substituted aromatic retinoids have also been shown to be effective as photoaffinity probes as the substituted retinoids retain their biological activity and affinity for CRBP [58].

Recently, RBP has been reported to have a role in adiposity, insulin resistance, and type 2 diabetes [59,60,61,62]. It would be interesting to further analyse these newly discovered functional roles of RBP by photoaffinity labelling technique. There has not been any report on photolabeling RBP, but retinoic acid should be a useful probe for this purpose since RBP binds retinoic acid at similar affinity as retinol [51,63]. A retinol azido-derivative, 4-azidoretinol, has been reported to be displaced by all-*trans*-retinol in labelling CRBP from liver, which should also be applicable to photolabel RBP [64].

### 2.5. Vitamin D-Binding Protein (Gc-Globulin)

**Figure 9:**
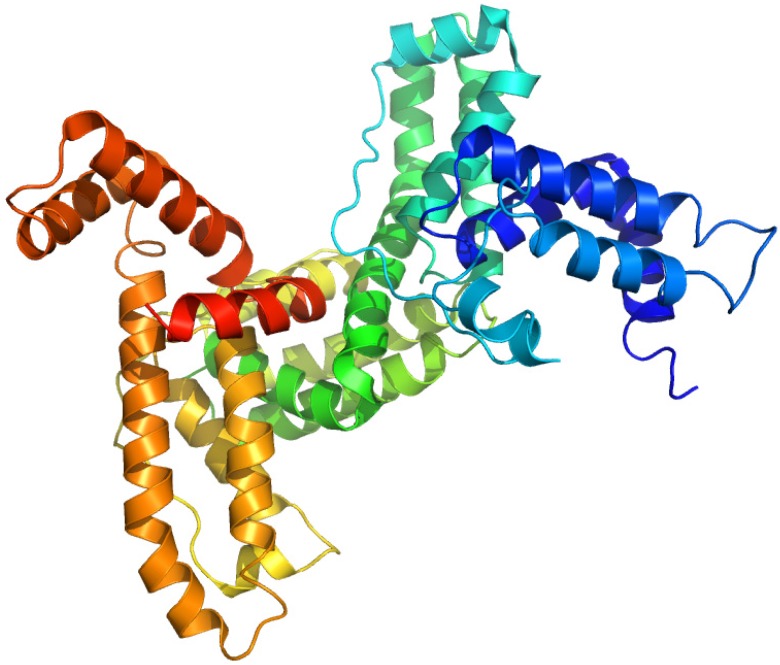
Crystal structures of the vitamin D-binding protein. The illustration was produced by PyMol using the atomic coordinates from the Protein Data Bank, PDB1J78 [68].

Vitamin D (cholecalciferol) is carried in the bloodstream to the liver, where it is hydroxylated by the microsomal enzyme vitamin D 25-hydroxylase to form 25-hydroxycholecalciferol (calcidiol). (Figure 9) Circulating calcidiol is then released into the plasma, where it binds to an α-globulin, vitamin D binding protein (VDBP). The resulting 25(OH)D_3_ is the major circulating form of vitamin D. It is then transported to the proximal tubules of the kidneys, where it is hydroxylated again at either the 1-α or the C24 position to form calcitriol (1,25-dihydroxycholecalciferol, 1α,25(OH)_2_D) or 24R25(OH)_2_D_3_. Calcitriol, which is the physiologically active form of vitamin D, is released into the circulation and transported to various target organs by binding to VDBP [65].

VDBP is a member of the albumin gene family, and a multifunctional single chain plasma protein that is predominantly synthesized in liver with structural and functional properties similar with serum albumin, afamin and α-fetoprotein. It is found in plasma, ascitic fluid, cerebrospinal fluid and on the surface of many cell types. It binds to vitamin D and its plasma metabolites and transports them to target tissues and transports fatty acids as well as monomeric actin. VDBP has been shown to play a role in the systemic control of osteoclastic bone resorption, and behave as a co-chemotaxin specific for the complement peptide C5a. Its galactose or sialic acid-free form has been reported to be potent macrophage-activating factors [66,67].

#### 2.5.1. 1,25-Dihydroxy-[26,27-^3^H]vitamin D_3_

Vitamin D_3_ undergoes photoisomerisation to form suprasterol I, suprasterol II and 5,6-transvitamin D_3_ [69]. Link *et al*. reported that 25-OH-[26,27-^3^H]vitamin D_3_ was capable of forming covalent bonds with human plasma proteins upon photoirradiation [70]. Underivatized 1,25-dihydroxy[26,27-^3^H]vitamin D_3_ was successfully used to photolabel the 1,25-dihydroxyvitamin D_3_ receptor upon ultraviolet irradiation at 220–280 nm [71,72].

#### 2.5.2. 3-Deoxy-3-azido-25-hydroxyvitamin D_3_

3-Deoxy-3-azido-25-hydroxyvitamin D_3_ (Az-25-OH-D_3_) is synthesized from tritiated 25-hydroxy-vitamin D_3_ by the direct attachment of the photoactivatable azido substituent to the C-3 position, as C-3 does not seem to involve in ligand binding. Az-25-OH-D_3_ showed only a 20-fold reduction in binding affinity compared to the parent compound, and becomes covalently bound to VDBP upon photoirradiation with medium-intensity, short-wavelength (254-nm) UV light for 5 min or less. The covalent binding was inhibited by 70%–80% in the presence of 25-hydroxyvitamin D_3_, indicating the covalent binding took place at the vitamin D binding site of the protein. Four plasma proteins were photolabeled by Az-25-OH-D_3_ when photolabeling experiment was conducted using human plasma. VDBP was the only protein of the four that was unlabeled in the presence of 25-hydroxyvitamin D_3_ [70].

#### 2.5.3. 25-Hydroxy(26,27-^3^H)vitamin D_3_ -3β-3'-[N-(4-azido-2-nitrophenyl)amino]propyl Ether

25-Hydroxyvitamin D_3_ 3β-[N-(4-azido-2-nitrophenyl)glycinate] (25-OH-D_3_-ANG), a photoaffinity analogue of 25-OH-D_3_ was shown by Ray *et al*. to specifically bind to rat VDBP and to covalently label the protein on UV photoirradiation for 15 min [73]. However, the vulnerability of the ester bond in ^3^H-25 OH-D_3_-ANG in the strongly basic pH conditions during carboxymethylation of the photolabeled protein led to a complete loss of the radioactivity, impeding further identification of the labelling site. A hydrolytically stable photoaffinity analogue of 25-OH-D_3_, 25-hydroxyvitamin D_3_ 3β-3'-[N-(4-azido-2-nitrophenyl)amino]propylether (25-OH-D_3_-ANE) was developed. A 2 min UV irradiation was found to be effective in covalently labeled the rat VDBP [74]. The cyanogen bromide fragment of the photolabeled rat VDBP with a molecular weight of 11.5 kDa that represents the N-terminus through residue 108 of the intact protein was found to contain most of the covalently attached radioactivity [75]. The 25-OH-D(3)-binding pocket of the vitamin D-binding protein appears to be sterically restricted [76]. Further photolabeling experiments using photoaffinity analogues of 25-hydroxyvitamin D_3_ with probes at the C-19 position that failed to specifically label VDBP shed light on the orientation of 25-OH-D_3_ in the binding pocket in which the C3-OH group at the A-ring points towards the binding cavity, while the C19-exocyclic methylene group projects outside the cavity [77,78].

### 2.6. α-Fetoprotein

α-Fetoprotein (AFP) is thought to be the fetal counterpart of serum albumin. It is a major plasma protein during fetal life, but expression in adults is often associated with hepatoma or teratoma. α-Fetoprotein exists in monomeric, dimeric and trimeric forms. It binds copper, nickel, fatty acids and bilirubin [79].

#### 2.6.1. 16-Diazo[^3^H]estrone and 4-azido[^3^H]estradiol

16-Diazo[^3^H]estrone has been shown to form a covalent attachment at the estrogen-binding site of rat AFP upon photoirradiation for 30 min at 300 nm. The photocovalent attachment appears to be predominated by pseudophotoaffinity labelling. On the other hand, 4-azido[^3^H]estradiol has been reported to undergo extensive photocovalent attachment to AFP but the attachment is nonspecific in nature [80].

### 2.7. Sex Hormone-Binding Globulin

**Figure 10:**
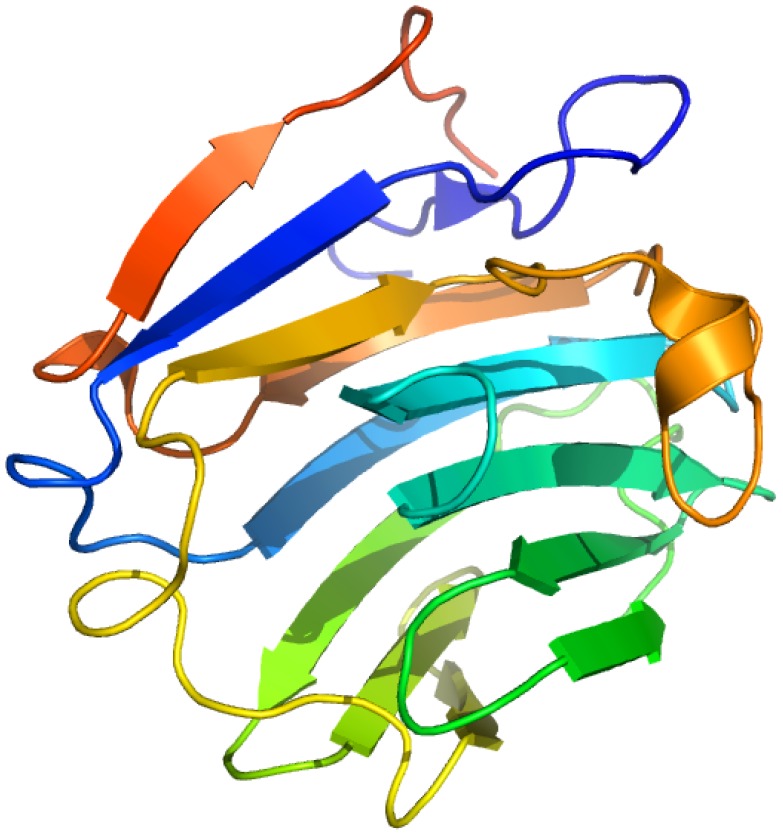
Crystal structures of the human sex hormone-binding globulin. The illustration was produced by PyMol using the atomic coordinates from the Protein Data Bank, PDB1KDK [83].

Sex hormone-binding globulin (SHBG) is a homodimeric plasma glycoprotein that participates in regulating steroid responses. (Figure 10) Human SHBG contains one O-linked and two N-linked oligosaccharides at Thr-7 and at Asn-351 and Asn-367, respectively, but the glycans do not seem to involve in steroid binding [81]. This protein has a high binding affinity for 17β-hydroxysteroid hormones such as testosterone and oestradiol. Plasma SHBG concentrations are affected by a number of different diseases as well as drugs, hence measuring SHBG levels can be useful in the evaluation of mild disorders and the identification of patients who are more likely to respond to therapy [82].

#### 2.7.1. [^3^H]Δ^6^-Testosterone ([1,2-^3^H]17β-hydroxy-4,6-androstadien-3-one) and Δ6-[17-^3^H] Estradiol

[^3^H]Δ^6^-Testosterone can be used as a photolabeling agent due to fact that the unsaturated ketone in the molecule is photoexcitable at 345 nm [84,85]. Hydrogen abstraction via a long lived excited triplet state upon photoirradiation has been proposed to be the covalent bond formation mechanism for both Δ^6^-testosterone and Δ^6^-[^3^H]estradiol [86]. The methionine at position 139 of human SHBG interacts with the photoaffinity ligand, Δ6-testosterone [87,88]. Purified rabbit and sheep SHBGs photolabeled by unsubstituted delta 6-testosterone revealed that the photoattachment occurred exclusively at methionine-133 of rabbit SHBG, whereas the methionine-139 residue is the main site of photolabeling in sheep SHBG [81]. Human SHBG purified from late-pregnancy serum and labelled with [^3^H]delta 6-testosterone, either by iodination (125I) or by photoaffinity were used to investigate the internalization of SHBG in the epididymis of the cynomologus monkey, *Macaca fascicularis* [89,90]. On the other hand, rat androgen-binding protein photolabelled with [^3^H]Δ^6^-testosterone has been used to study the binding of photolabelled ABP to plasma membranes of epithelial epididymal cells in rats [91]. In an attempt to further characterise the steroid binding site, Chambon *et al*. synthesized nine photoaffinity labelling reagents of 17α-alkylamine derivatives of 3β-androstanediol to conduct topological mapping. The study revealed that the conserved Trp-84, Pro-130 and Lys-134 residues are all located in the vicinity of the D ring of the steroid ligand binding site of human SHBG [92].

### 2.8. Corticosteroid-Binding Globulin

**Figure 11:**
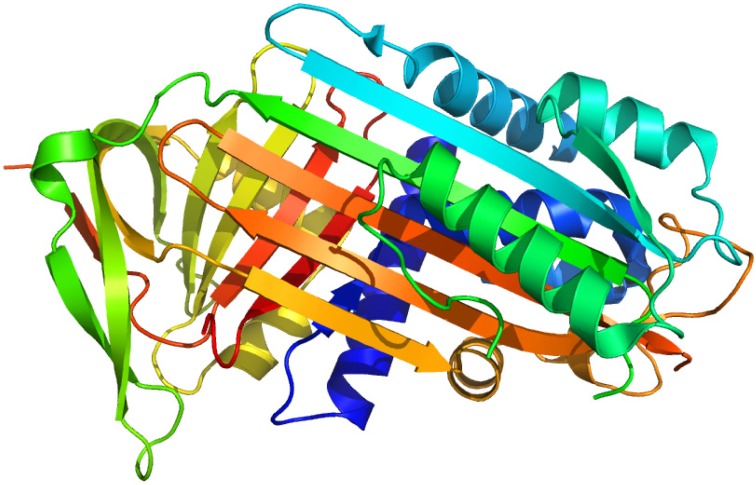
Crystal structures of the human corticosteroid-binding globulin. The illustration was produced by PyMol using the atomic coordinates from the Protein Data Bank, PDB2VDY [96].

Corticosteroid-binding globulin (CBG) is a member of the serine proteinase inhibitor (serpin) family that functions in the transport of glucocorticoids and progestins in the blood and regulates their access to target cells. (Figure 11) CBG is an α-globulin protein and a heavily glycosylated protein containing six N-linked glycans that are involved in the CBG-receptor interaction. Its steroid-binding site resembles the thyroxin-binding site in the related serpin, thyroxin-binding globulin [93,94,95].

#### 2.8.1. 21-Diazo-21-[6,7-^3^H]deoxycorticosterone and Unsubstituted Tritiated Δ^6^-derivatives of Cortisol, Corticosterone, Progesterone and Testosterone

21-Diazo-21-[6,7-^3^H]deoxycorticosterone was first synthesized as an analog of corticosterone of high specific activity, for photolabeling the high affinity steroid-binding human corticosteroid-binding globulin. Irradiation of CBG with the 21-diazo derivative resulted in irreversible binding to the protein [97]. A later attempt in specific covalent labelling of purified human CBG by 2-fold molar excess of unsubstituted Δ^6^-[^3^H]cortisol, Δ^6^-[^3^H] corticosterone, and Δ^6^-[^3^H]progesterone after 30 min irradiation at >300 nm produced a labelling efficiency of 0.21, 0.14, and 0.08 mol of label/mol of CBG respectively. The most plausible mechanism of photolabeling was preferential hydrogen abstraction by excited triplet radicals derived from the dienone moiety in these three compounds. Trp-371 was concluded to be the main site of specific covalent bond formation with the three steroids [98]. On the other hand, [^3^H]Δ^6^-testosterone has been shown to also form specific covalent binding with hamster serum CBG, in addition to human SHBG [99].

## 3. Conclusions

The advantages of using a natural ligand with a photoreactive group as a photolabeling agent include avoiding laborious chemical synthesis and the subsequent determination of the binding affinity of the derivatised photolabeling agent. The great advantage of using underivatized natural ligands for photoaffinity labeling lies not only in the fact that they are easily availabile but also because non-specific covalent binding is typically minimal, since the binding specificity of the natural photolabile ligand remains constant. This is especially important when the identification of binding protein in plasma that contains an abundance of different types of proteins might by chance interact with the derivatised photolabeling agent with a high affinity. Table 1 lists examples of natural ligands with a photoreactive moiety that could potentially be used to photolabel proteins. 

**Table 1:** Natural ligands with photolabile moieties.

Natural ligand with photoreactive moiety	Chemical structure	Photoirradiation wavelengths (nm) and time	Target protein	Reference
[^14^C]Halotane	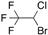	254 (10 s)	Albumin	[23]
[^3^H_8_]Leukotriene E_4_	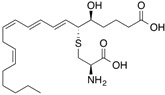	300 (4 min)	Albumin, glutathione transferases	[28]
[^14^C]Ketoprofen	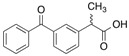	310 (30 min)	HSA	[11]
[^3^H]Flunitrazepam	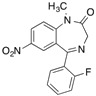	310 (30 min)	HSA, AGP, benzodiazepine receptor	[22,38]
[^3^H]7-Hydroxystaurosporine (UCN-01)	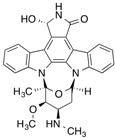	310 (30 min)	AGP	[41]
[^3^H]Glibenclamide	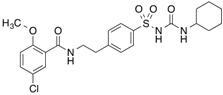	254 (3 min)	140 kDa sulfonylurea receptor in the rat beta-cell membrane and albumin	[100]
[^3^H]Benzylpenicillin	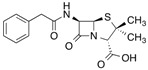	254 (2 min)	127 kDa brush border membrane protein from rabbit small intestine and albumin	[101]
[3',5'-^125^I]Thyroxine ([^125^I]T4), [3'-^125^I]Triiodothyronine	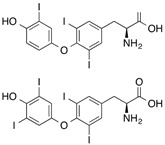	>300 (80 s)	Human thyroxine binding globulin and bovine serum albumin	[47]
All-trans-[11,12-^3^H]Retinoic acid	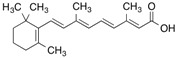	365 (15 min)	Cellular retinoic acid-binding protein and albumin	[56]
[^14^C]Doxorubicin	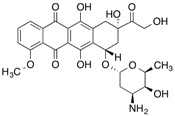	>254 (5 min)	Junctional sarcoplasmic reticulum	[102]
[^3^H]Taxol	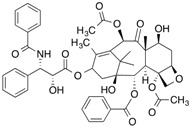	254 (30 min)	Tubulin	[103]
[5-^3^H]Dolastatin 10	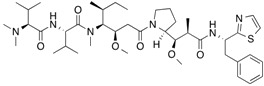	254nm (10 min)	Beta-tubulin	[104]
[^3^H]Colchicine	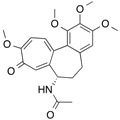	>305 (5 min)	Tubulin	[105]
[^3^H]Vinblastine	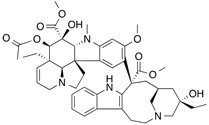	>310 (7 min)	Tubulin	[104]
[^3^H]Forskolin	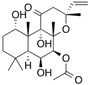	254 (30 s)	Erythrocyte D-glucose transporter	[106]
[^3^H]Cytochalasin B	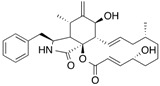	254 (30 s)	Human erythrocyte glucose transporter	[107]
[^3^H]Nitrendipine	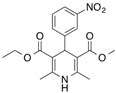	254 (20 s)	32 kDa cardiac membrane protein	[108]
[^3^H]Bumetanide	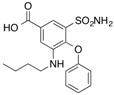	Flash lights (2 min) 300–400 (10 min)	Na^+^-K^+^-Cl^−^ cotransporter	[109,110]
[^3^H]Clonazepam	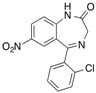	Shortwave UV (60 min)	Central type of benzodiazepine receptor	[111]

In addition, the identification of the binding proteins in plasma would assist in the accurate characterization of the pharmacokinetic profiles of drugs [17,112]. Emerging new high-throughput analytical technologies would undoubtedly extend the potential of photoaffinity labeling to become a rapid with high sensitivity and robust means for target protein identification [1].
